# Design Strategies of Li–Si Alloy Anode for Mitigating Chemo‐Mechanical Degradation in Sulfide‐Based All‐Solid‐State Batteries

**DOI:** 10.1002/advs.202301381

**Published:** 2023-06-26

**Authors:** Minhyung Kim, Min Ju Kim, Yeong Seon Oh, Sung Kang, Tae Ho Shin, Hyung‐Tae Lim

**Affiliations:** ^1^ Department of Materials Convergence System Engineering Changwon National University Changwon Gyeongnam 51140 Republic of Korea; ^2^ Analysis and Assessment Center Research Institute of Industrial and Science Technology Pohang Gyeongbuk 37673 Republic of Korea; ^3^ Hydrogen Energy Materials Center Korea Institute of Ceramic Engineering and Technology Jinju 52851 Republic of Korea; ^4^ School of Materials Science and Engineering Changwon National University Changwon Gyeongnam 51140 Republic of Korea

**Keywords:** all‐solid‐state batteries, composite structures, cutoff voltage, Li–Si alloys, mechanical failures, redox activities, sulfide electrolytes

## Abstract

Composite anodes of Li_3_PS_4_ glass+Li–Si alloy (Type 1) and Li_3_N+LiF+Li–Si alloy (Type 2) are prepared for all‐solid‐state batteries with Li_3_PS_4_ (LPS) glass electrolyte and sulfur/LPS glass/carbon composite cathode. Using a three‐electrode system, the anode and cathode potentials are separated, and their polarization resistances are individually traced. Even under high‐cutoff‐voltage conditions (3.7 V), Type 1 and 2 cells are stably cycled without voltage noise for >200 cycles. Although cathode polarization resistance drastically increases after 3.7 V charge owing to LPS oxidation, LPS redox behavior is fairly reversible upon discharge–charge unlike the non‐composite alloy anode cell. Time‐of‐flight secondary ion mass spectrometry analysis reveals that the enhanced cyclability is attributed to uniform Li–Si alloying throughout the composite anode, providing more pathways for lithium ions even when these ions are over‐supplied via LPS oxidation. These results imply that LPS‐based cells can be reversibly cycled with LPS redox even under high‐cutoff voltages, as long as non‐uniform alloying (lithium dendrite growth) is prevented. Type 1 and 2 cells exhibit similar performance and stability although reduction product is formed in Type 1. This work highlights the importance of alloy anode design to prevent chemo‐mechanical failure when cycling the cell outside the electrochemical stability window.

## Graphical abstract

All‐solid‐state batteries (ASSBs) can chemo‐mechanically fail owing to redox activities of sulfide electrolytes. Li_3_PS_4_ (LPS)‐based ASSBs with a composite structured Li–Si alloy anode are reversibly cycled, even outside the electrochemical stability window. This enhanced cyclability is attributed to uniform Li–Si alloying throughout the anode, providing more pathways for Li ions, even when these ions are over‐supplied via LPS oxidation.

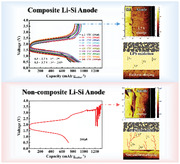

## 1 Introduction

All‐solid‐state batteries (ASSBs) are promising next‐generation energy storage devices to replace conventional lithium‐ion batteries because of their high energy density, simple fabrication, and improved safety.^[^ ^1^, ^2^, ^3^, ^4^, ^5^, ^6^, ^7^ ^]^ Over a few decades, inorganic solid electrolytes (SEs) have been extensively developed to achieve ionic conductivities as high as those of conventional liquid electrolytes.^[^ ^8^, ^9^ ^]^ The inorganic SEs are mainly sulfides (Li–P–S‐based glass, glass ceramics [*x*Li_2_S⋅[1−*x*]P_2_S_5_], thio‐LISICON [Li_3.25_Ge_0.25_P_0.75_S_4_], Li_10_GeP_2_S_12_ [LGPS], and argyrodite [Li_6_PS_5_X, X = Cl, Br, I]) and oxides (perovskite oxide [[Li,La]TiO_3_], NASICON‐type [LiTi[PO_4_]_3_], and garnet‐type [Li_7_La_3_Zr_2_O_12_]).^[^ ^10^, ^11^, ^12^ ^]^

Each type of inorganic SEs has its advantages and drawbacks. In terms of grain boundary conductivity (related to conductivity at room temperature) and feasible battery construction (related to SE/electrode interfacial resistance), sulfide SEs are superior to oxide SEs owing to their lower electronegativity and more ductile mechanical properties.^[^ ^13^, ^14^, ^15^, ^16^, ^17^, ^18^, ^19^, ^20^, ^21^, ^22^ ^]^ However, several bottlenecks hinder the commercialization of sulfide SE‐based ASSBs. For example, these SEs can react with ambient moisture to generate H_2_S, a problem that can be ameliorated by forming a novel core–shell structure in sulfide SEs and using zeolites as H_2_S scavengers.^[^ ^23^, ^24^, ^25^ ^]^ Moreover, most sulfide SEs have a narrow electrochemical stability window (ESW, 1.7–2.1 V vs Li/Li^+^), and they can react with high‐ and low‐voltage electrodes to form high‐resistance interphases.^[^ ^26^, ^27^, ^28^, ^29^ ^]^ The proposed strategies to prevent undesirable interactions due to the narrow ESW include metal cation doping and incorporation of LiI in sulfide SEs to stabilize the anode side, and surface coating with ion‐conducting oxides (Li_4_TiO_5_ and LiNbO_3_) to stabilize the cathode side.^[^ ^28^, ^30^, ^31^, ^32^, ^33^ ^]^ Moreover, sulfide SEs in ASSBs can be reduced and oxidized (as active materials) during the battery discharge/charge processes, which contributes charges to the total capacities but eventually reduces the cell performance.^[^ ^34^, ^35^ ^]^ Recently, their redox activity and behavior have been extensively studied under various experimental conditions.^[^ ^30^, ^36^, ^37^, ^38^, ^39^, ^40^, ^41^, ^42^, ^43^, ^44^, ^45^ ^]^ A highly important finding is that the decomposition kinetics of sulfide SEs strongly depends on the number of electronic pathways in the working electrode: a higher specific surface area of the carbon conductive agent promotes the SE decomposition kinetics.^[^ ^38^, ^44^ ^]^ Dewald et al. reported that an SE can be reversibly reduced and oxidized outside the theoretically (thermodynamically) predicted window, depending on the composition and transport properties of the working electrode, that is, a practical stability window exists for each electrode condition.^[^ ^42^ ^]^ The magnitude of the applied potential, that is, the cutoff‐voltage condition, also greatly influences the cyclability and degradation rates of sulfide‐based ASSBs. For example, a higher degradation rate was observed in a half‐cell of Li|Li_7_P_3_S_11_|(Li_7_P_3_S_11_+carbon) when cycled in a wider voltage range (0.5–3.5 V vs Li/Li^+^).^[^ ^39^ ^]^ Similarly, it was reported that in a Li–S cell based on Li_6_PS_5_Cl, reductive and oxidative degradation became significant at the potential conditions below 0.4 V and above 2.0 V (vs In/InLi), respectively.^[^ ^40^ ^]^ Therefore, analyzing the practical redox stability of sulfide SE‐based cells and determining the practical voltage window with respect to the electrode conditions are essential to secure their long‐term cyclability. However, most reported redox studies were carried out on non‐practical configuration cells, such as (SE+carbon)|SE|(SE+carbon) and (Li or Li–In)|SE|(SE+carbon), and aimed at a fundamental understanding of the redox behavior of sulfide SEs.^[^ ^36^, ^37^, ^38^, ^39^, ^42^, ^44^, ^45^ ^]^ To the best of our knowledge, only a few studies considered the redox activity of sulfide SEs in a practical configuration, namely (sulfur+carbon+Li_6_PS_5_Cl)|Li_6_PS_5_Cl|(Li–In),^[^ ^40^, ^41^ ^]^ (Li_2_S+carbon+Li_5.5_PS_4.5_Cl)| Li_5.5_PS_4.5_Cl|(Li–In),^[^ ^43^ ^]^ and (Li_2_S+carbon+LPS)|LPS|(Li–In), where LPS = Li_3_PS_4_.^[^ ^46^ ^]^

Researchers recently addressed the mechanical failure of sulfide‐based ASSBs, which originates from dendrite growth and loss of stack‐pressure control, as an important issue especially with lithium–metal or lithium–alloy anode.^[^ ^47^, ^48^, ^49^, ^50^, ^51^, ^52^, ^53^ ^]^ Our previous study reported that chemo‐mechanical failure can be induced by redox activity of the LPS glass SE, that is, oxidative decomposition of the SE induces chemo‐mechanical failure in a full cell with the configuration of (sulfur+carbon+LPS)|LPS|(Li–Si).^[^ ^54^ ^]^ We revealed that cracks and micro‐short circuits could develop through the SE by lithium dendrite growth on the Li–Si anode when lithium ions are over‐supplied via SE oxidation in the cathode under high‐cutoff‐voltage conditions. In addition to the deterioration of the working electrode, the sulfide SE's redox activity also affects the chemo‐mechanical stability of the entire cell.

To mitigate the chemo‐mechanical failure in Li–S ASSBs, in the present study, we used an alloying design strategy to fabricate two types of composite‐structured Li–Si alloy anodes: LPS glass SE + Li–Si alloy (denoted as Type 1) and (Li_3_N+LiF) SE + Li–Si alloy (denoted as Type 2). On the one hand, Li_3_N has a high ion conductivity and good chemical stability with Li metal but a low interface energy with Li metal. On the other hand, LiF has a high interface energy with Li metal and low electronic conductivity but a relatively lower ionic conductivity compared with Li_3_N.^[^ ^55^ ^]^ By combining Li_3_N and LiF in a composite, we can realize the advantages of both materials (high stability against Li metal, high ionic conductivity, and low electronic conductivity) to suppress Li dendrite growth. This composite‐type electrode structure has not been utilized in the alloy anodes of ASSBs, as conventional anodes in ASSB consist of lithium metal‐based alloys without SE.^[^ ^56^ ^]^ A few recent studies utilized composite anodes consisting of SE and a single metal that forms an alloy with lithium after the first charge, instead of SE and a lithium alloy to start with.^[^ ^57^, ^58^ ^]^ In the case of a non‐composite structure, lithium alloying (or plating) tends to occur at specific sites near the interface between the SE and alloy anode layers. In contrast, a composite structure results in evenly distributed reactive sites throughout the anode. To investigate and compare the effects of Type 1 and 2 composite anodes on the electrochemical performance and stability of Li–S ASSBs, we applied a three‐electrode system to bulk‐type cells consisting of a sulfur composite cathode and LPS glass SE. Cycling tests and impedance spectral analysis were conducted on the three‐electrode cell, so that the cathode and anode potentials were separated and their polarization‐resistance changes were individually traced. Moreover, two‐electrode (standard type) cells were constructed for long‐term cycle tests. Material analysis using X‐ray diffraction (XRD), scanning electron microscopy (SEM) with energy dispersive spectroscopy (EDS), and time‐of‐flight secondary ion mass spectrometry (TOF‐SIMS) with an ion milling system (IMS) was also conducted to reveal the effects of composite‐structured alloy anodes on the chemo‐mechanical stability.

## 2 Results and Discussion

Figures S1a and S1b, Supporting Information, show the XRD patterns of mixed Li_2_S and P_2_S_5_ powders before and after planetary ball milling (370 rpm, 30 h), respectively. All crystalline peaks corresponding to Li_2_S disappeared after milling, indicating the formation of LPS glass.^[^ ^59^ ^]^ An LPS disk pellet was prepared, and its impedance spectrum was measured using stainless‐steel rods at room temperature. The results (Figure S1c, Supporting Information) indicated a bulk resistance of ≈121 Ω, which corresponds to an ionic conductivity of ≈5.39 × 10^−4^ S cm^−1^. This is similar to the values previously reported by us and other researchers.^[^ ^54^, ^60^ ^]^ Figure S1d, Supporting Information, shows the XRD pattern of the composite cathode powder after ball milling, suggesting that sulfur changed from a crystalline phase to an amorphous one.^[^ ^61^, ^62^ ^]^ Figure S2a, Supporting Information, shows the XRD patterns of Li_13_Si_4_ alloy anode powder obtained after mechanical alloying, and Figures S2b–d, Supporting Information, show, respectively, the reference patterns of Li_13_Si_4_, Li, and Si from the International Centre for Diffraction Data database. The XRD results indicate the metastable phase of Li_13_Si_4_ was formed after mechanical alloying.^[^ ^63^ ^]^ XRD analysis was also conducted on Type 1 composite anode powder (Figure S3, Supporting Information), indicating that Li_2_S was formed as a secondary phase owing to reductive decomposition in the reaction between Li–Si alloy (an electronic conductor with a narrow band gap of *E*_g_ = 0.057 eV at *T* < 450 K and a low electrode potential of ≈0.22 V vs Li/Li^+^) and LPS (with a narrow ESW).^[^ ^38^ ^]^ Note that another possible reduction product (Li_3_P) was not detected by XRD.^[^ ^58^ ^]^ The XRD patterns of as‐received Li_3_N, as‐received LiF, composite powder of Li_3_N and LiF after ball milling, and Type 2 composite anode powder after ball milling are shown in Figures S4a–d, Supporting Information, respectively. According to Figure S4c, Supporting Information, no secondary phases were formed during the preparation of Type 2 composite anode powder, and only peaks corresponding to the *β*‐Li_3_N and LiF phases were present, indicating that the *α*‐Li_3_N phase was converted to the *β*‐Li_3_N phase after the first ball milling (Li_3_N+LiF), as reported previously.^[^ ^55^ ^]^ However, a peak corresponding to the *α*‐Li_3_N phase was detected again after the second ball milling (Li_3_N+LiF+Li_13_Si_4_), as shown in Figure S4d, Supporting Information.

**Figure 1:**
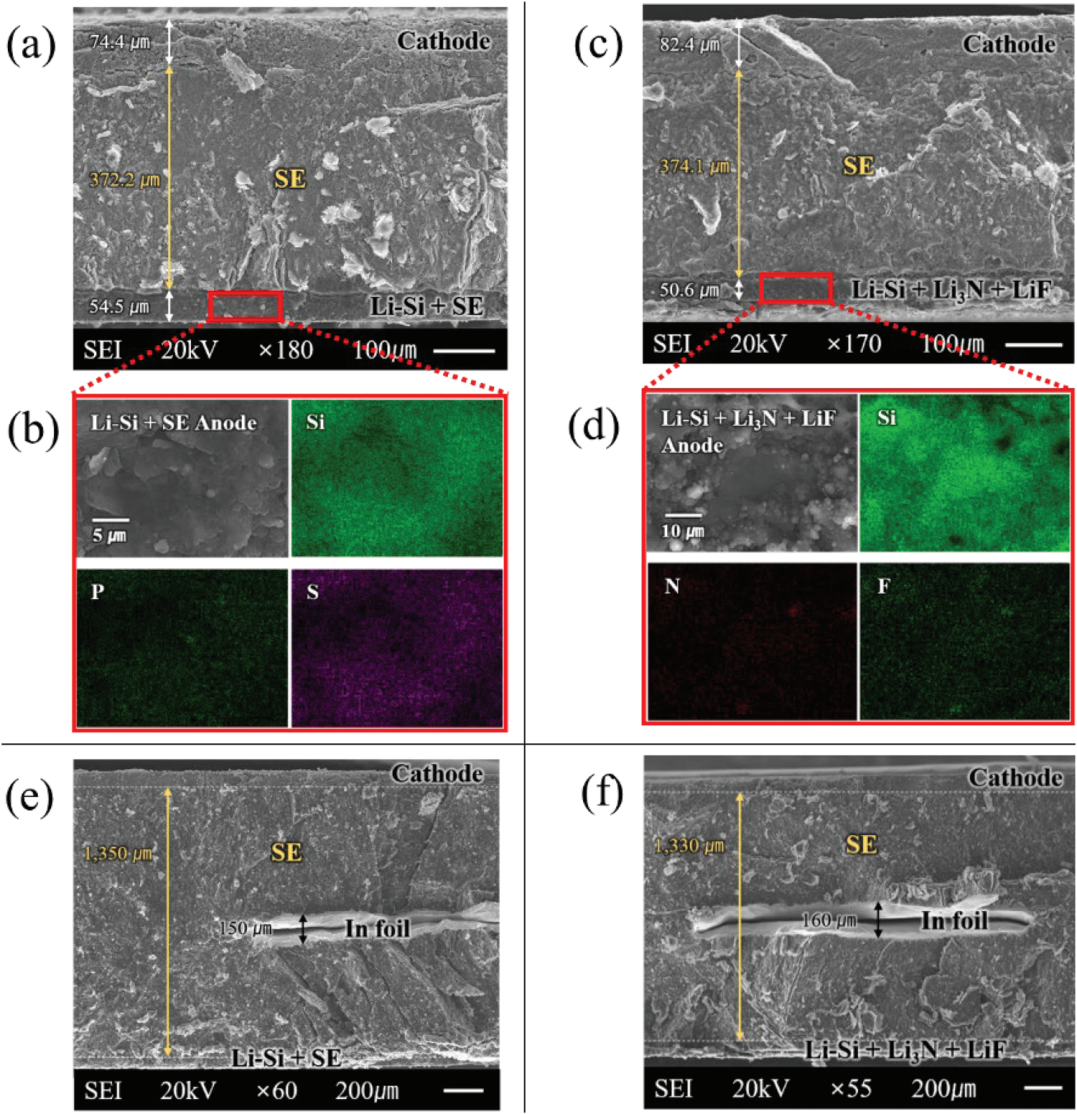
a) Cross‐sectional SEM image (×180) of the three layers (cathode, SE, and anode) in the standard T1 cell. b) SEM image (×4300) of Type 1 composite anode and EDS mapping results of Si, P, and S. c) Cross‐sectional SEM image (×170) of the three layers (cathode, SE, and anode) in the standard T2 cell. d) SEM image (×4300) of Type 2 composite anode and EDS mapping results of Si, N, and F. Cross‐sectional SEM images of the three‐electrode e) T1 cell and f) T2 cell.

**Figure** **1**a shows the cross‐sectional SEM image (×180) of three layers in the fabricated standard T1 cell, with the cathode, SE, and Type 1 composite anode layers having thicknesses of ≈75, ≈370, and ≈55 µm, respectively. Figure 1b shows the SEM image (×4300) of Type 1 composite anode layer in addition to the EDS mapping results of Si, P, and S. The Li–Si alloy and LPS particles were evenly distributed throughout the Type 1 composite anode. Similarly, Figure 1c shows the cross‐sectional SEM image (×170) of three layers in the fabricated standard T2 cell, with the cathode, SE, and Type 2 composite anode layers having thicknesses of ≈82, ≈374, and 24 µm, respectively. Figure 1d shows a magnified SEM image (×2200) of the Type 2 composite anode layer in addition to the EDS mapping results of Si, N, and F. Li–Si and Li_3_N+LiF composite were evenly distributed in the Type 2 composite anode. Figures 1,f show the cross sections of fabricated three‐electrode T1 and T2 cells, respectively. In both cells, the total thickness of the SE layer with embedded In foil as RE was confirmed to be ≈1350 µm. Note that the thickness of the three‐electrode cells was set to be 3–4 times larger than that of the standard cells to prevent physical contact (short circuit) between the RE and working electrodes (cathode and anode). Later in this paper, we will prove that this thickness did not significantly affect the electrochemical performance (discharge–charge capacities) of the three‐electrode cells at a low current of 200 µA.

**Figure 2:**
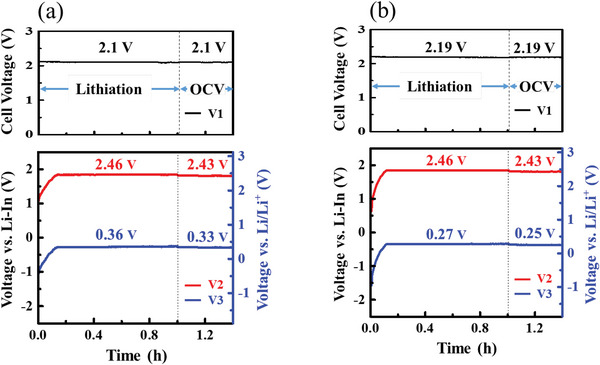
V1, V2, and V3 profiles of three‐electrode a) T1 and b) T2 cells during lithiation on RE and under open‐circuit conditions after lithiation.

**Figure 3:**
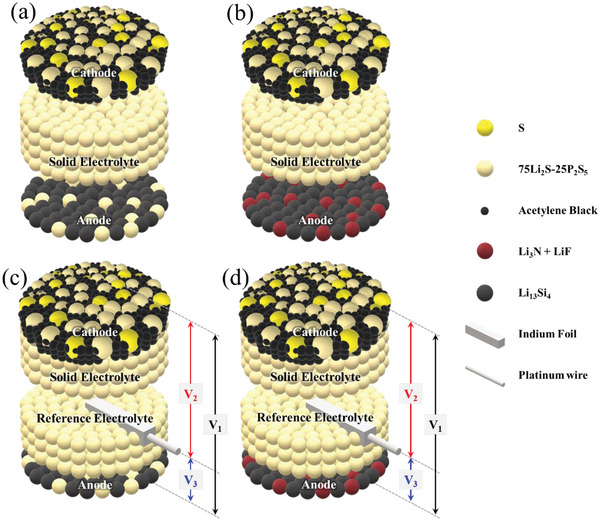
Schematics of the fabricated two‐electrode (standard) a) T1 and b) T2 cells and three‐electrode c) T1 and d) T2 cells.

Before testing the three‐electrode T1 and T2 cells, the embedded In foil (RE) was lithiated by applying a constant current of 20 µA for ≈40 min between the RE and composite anode. **Figures** **2**a,b show the three voltage profiles (V1, V2, and V3 as defined later in **Figure** **3** in Experimental Section) for the T1 and T2 cells, respectively, both during RE lithiation and under open‐circuit conditions after lithiation. The Y1 and Y2 axes of the bottom figures indicate the electrode potential versus RE and Li/Li^+^, respectively, and the inserted V1–V3 values in the figures correspond to Y2. Upon lithiation, V2 and V3 immediately matched the applied current, and then they stabilized at ≈2.46 and ≈0.36 V for T1 cell and ≈2.46 and ≈0.27 V for T2 cell, respectively. The stabilized V2 value is identical between T1 and T2 cells, but the stabilized V3 values are slightly different owing to reduction products formed in T1 cell (as confirmed by XRD results in Figure S3, Supporting Information). Figure S5, Supporting Information, shows the three voltage profiles for the T0 cell during and after lithiation, and both the V2 and V3 values are similar to those of T2 cell. In the T0 cell, the SE layer might also react with the Li–Si alloy, but the interfacial area between the LPS and Li–Si alloy was significantly smaller than that of the T1 cell owing to the layer/layer interface. Therefore, decomposition kinetics in the T0 cell should be sluggish. The reduction product of LPS (Li_2_S) has a high electronic resistance.^[^ ^52^, ^55^ ^]^ It was reported that the solid electrolyte interphase (SEI) layer formed by the reduction products of Li_6_PS_5_Cl SE, namely Li_2_S (insulating), Li_3_P (ion‐conductive), and LiCl, functions as “a passivating layer” that can prevent undesirable continuous reactions between Li–Si and SE,^[^ ^58^ ^]^ as shown in Figure S6, Supporting Information.

A cell consisting of (Li_7_Si_3_ + LPS) | LPS | (Li_13_Si_4_+LPS) was prepared to investigate the interface stability of LPS SE and Type 1 anode. The (Li_7_Si_4_+LPS) electrode was lithiated using a current of 20 µA for ≈5 h to achieve approximately the same electrode potential (corresponding to the phases of Li_13_Si_4_ + Li_7_Si_3_) for both the electrodes, that is, to obtain the cell voltage close to 0 V. Subsequently, this symmetric cell was discharged and charged at 0.26 mA cm^−2^ for ≈120 h, as shown in Figure S7, Supporting Information. A capacity of 2 mAh cm^−2^ was used per cycle. We can observe the stable cycling performance in this symmetric cell, indicating the good interface stability at LPS SE/Type 1 anode. Later in this paper, we will discuss the effect of reductive decomposition products of Type 1 composite anode on cell stability during long‐term cycling tests of the standard cell.

**Figure 4:**
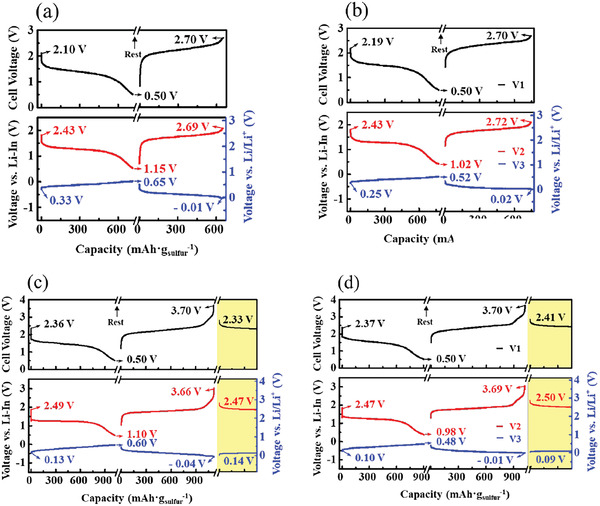
V1, V2, and V3 profiles of the three‐electrode a) T1 and b) T2 cells for the initial discharge–charge in the voltage range of 0.5–2.7 V. V1, V2, and V3 profiles of the c) T1 and d) T2 cells for the second discharge–charge in the voltage range of 0.5–3.7 V.

**Figures** **4**a,b show the three voltage profiles for the T1 and T2 cells, respectively, during the initial discharge–charge at a constant current of 200 µA in the cell voltage range of 0.5–2.7 V. At the end of the first discharge, the values of V2 and V3 are 1.15 and 0.65 V in the T1 cell and 1.02 and 0.52 V in the T2 cell, respectively. At the end of the first charge, these voltages are 2.69 and −0.01 V in the T1 cell and 2.72 and 0.02 V in the T2 cell, respectively. Note that these voltages are relative to Li/Li^+^. The V3 profiles of T1 and T2 cells are equivalent to those of half cells consisting of Li–In | LPS | composite anode. Based on the measured V3 profiles and the calculated quantity of electric charge during (dis)charge, we can understand that the phases of both Type 1 and 2 anodes during discharge–charge correspond to Li_13_Si_4_+Li_7_Si_3_. For both cell types, V2 and V3 during the discharge–charge cycle exceeded the upper and lower limits of the theoretical ESW of LPS. Our previous study demonstrated that T0 cells were reversibly discharged–charged for up to ≈80 cycles with similar behavior in the three voltages under the same cutoff‐voltage condition.^[^ ^54^ ^]^ Additionally, similar results were reported by other research groups.^[^ ^41^, ^42^ ^]^ Therefore, we can consider this cutoff‐voltage condition a practical ESW for LPS‐based cells. After two cycles in the cell voltage range of 0.5–2.7 V, the charge cutoff voltage was increased to 3.7 V for both T1 and T2 cells. Figures 4c,d show the three voltage profiles for the T1 and T2 cells, respectively, during the second discharge–charge (at 200 µA) in the cell‐voltage range of 0.5–3.7 V. On the one hand, after completing discharge at the cell voltage of 0.5 V and charging to 3.7 V, the V2 values of T1 and T2 cells were extended to 3.66 and 3.69 V, respectively, which are considerably higher than those obtained at 2.7 V charge. On the other hand, the V3 values of T1 and T2 cells did not significantly change upon increasing the cutoff voltage to 3.7 V. After completing the 3.7 V charge, the three voltage profiles were measured under open‐circuit conditions (yellow boxes in Figure 4c,d), with V2 = 2.47 V and V3 = 0.14 V for T1 cell and V2 = 2.50 V and V3 = 0.09 V for T2 cell. On the one hand, both V3 values are lower than those measured after lithiation (see Figure 2a,b), which might be due to alloying beyond the composition of Li_13_Si_4_ and/or Li plating in the composite anode. On the other hand, the V2 values of both T1 and T2 cells did not significantly change after the 3.7 V charge although LPS in the cathode was oxidatively decomposed during the 3.7 V charge, as confirmed previously by X‐ray photoelectron spectroscopy.^[^ ^54^ ^]^ Figure S8, Supporting Information, shows the three voltage profiles of the T0 cell during charging to 3.7 V. V2 and V3 changed normally with time until the cell voltage was ≈3.0 V, beyond which both exhibited continuous noise, indicating the formation of a micro‐short circuit through the SE via Li dendrite growth on the non‐composite Li–Si alloy anode.^[^ ^54^ ^]^ These voltage behaviors and signs of short circuit were not observed in T1 and T2 cells.

**Figure 5:**
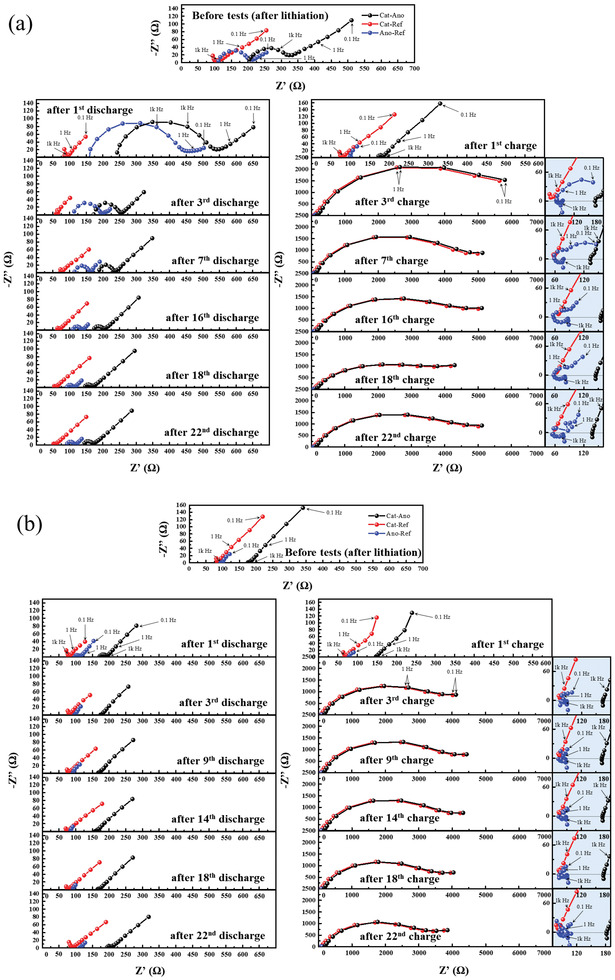
Impedance spectra of the three‐electrode a) T1 and b) T2 cells measured before and during discharge–charge tests. Note that the first two cycles were conducted in the cell‐voltage range of 0.5–2.7 V, and the other cycles were conducted in 0.5–3.7 V.

During cycling tests of the three‐electrode T1 and T2 cells, impedance spectra were frequently measured (**Figures** **5**a,b, respectively) to trace changes in the polarization resistance of each electrode. The impedance spectra of the cathode–anode full cell, the cathode–RE half‐cell, and the anode‐RE half‐cell are denoted as “Cat‐Ano,” “Cat‐Ref,” and “Ano‐Ref,” respectively. The first two cycle tests were conducted in the cell voltage range of 0.5–2.7 V, and then in the range of 0.5–3.7 V. No significant difference was observed between the impedance spectra of T1 and T2 cells, except for the initial large semicircle in “Ano‐Ref” of T1 cell that can be explained by an SEI layer of reduction products formed on the Type 1 composite anode (as confirmed in Figure S3, Supporting Information). Note that the semicircle size of “Ano‐Ref” in T1 cell increased with discharging (de‐alloying) and decreased with charging (alloying), which might be due to Li concentration change and void formation–densification in the anode during discharge–charge.^[^ ^58^ ^]^ Furthermore, the semicircle of “Ano‐Ref” of T1 cell became increasingly smaller with cycling, especially in the discharged state, which might be due to improvements in the morphology and microstructure of the alloy anode. Note that the same behaviors of “Ano‐Ref,” namely 1) increasing with discharging and decreasing with charging and 2) decreasing gradually with repeated cycles, were also observed in T2 cell although the initial size of “Ano‐Ref” was considerably small. To confirm whether the large semicircle of “Ano‐Ref” was due to the reduction product Li_2_S, we prepared another three‐electrode cell with a composite anode consisting of Li_13_Si_4_ and Li_2_S (80:20 wt%), denoted as T1‐1 cell. Figure S9a, Supporting Information, shows the three voltage profiles of T1‐1 cell during lithiation. The values of V2 and V3 are similar to those of T1 cell under open‐circuit conditions after lithiation. Figure S9b, Supporting Information, shows changes in the impedance spectra of “Cat‐Ano,” “Cat‐Ref,” and “Ano‐Ref” for T1‐1 cell during cycling. The first two cycles were conducted under the 0.5–2.7 V condition, and then cycling was continued under the 0.5–3.7 V condition. Similar to the T1 cell, the semicircle of “Ano‐Ref” of T1‐1 cell was initially large and gradual decreased with cycling, indicating that the T1‐cell behavior is mainly due to the reduction product Li_2_S formed in the alloy anode. Note that this large initial anode polarization resistance (caused by LPS decomposition) of the T1 cell did not substantially affect its gravimetric capacity or overall performance compared with the T2 cell, which was confirmed in the standard cell tests in this study. When the cells were charged up to 3.7 V from the third cycle on, the semicircle in “Cat‐Ref” drastically increased in both T1 and T2 cells owing to the oxidation of LPS in the cathode, but it recovered to the initial level with discharging. As the cycles were repeated, the semicircle of “Cat‐Ref” in the 3.7 V charged state became slightly smaller in both T1 and T2 cells. This behavior indicates that LPS in the cathode was reversibly oxidized and reduced during cycling in the voltage range of 0.5–3.7 V, in sharp contrast to the T0 cell that shows voltage noise and irrecoverable impedance in “Cat‐Ref” even with discharging (Figures S8 and S10, Supporting Information).^[^ ^54^ ^]^ As LPS in the cathode was oxidized during charging to 3.7 V, Li dendrite growth was induced and short circuit occurred in the SE (as reflected in the voltage noise in Figure S8, Supporting Information), leading to permanent cathode deterioration as reflected by the irreversible increase in “Cat‐Ref” in Figure S10, Supporting Information.^[^ ^54^ ^]^ The T0 and T1/T2 cells only differed in the structure of their anode (non‐composite vs composite), implying that short‐circuit formation and mechanical failure were avoided owing to the composite structure.

**Figure 6:**
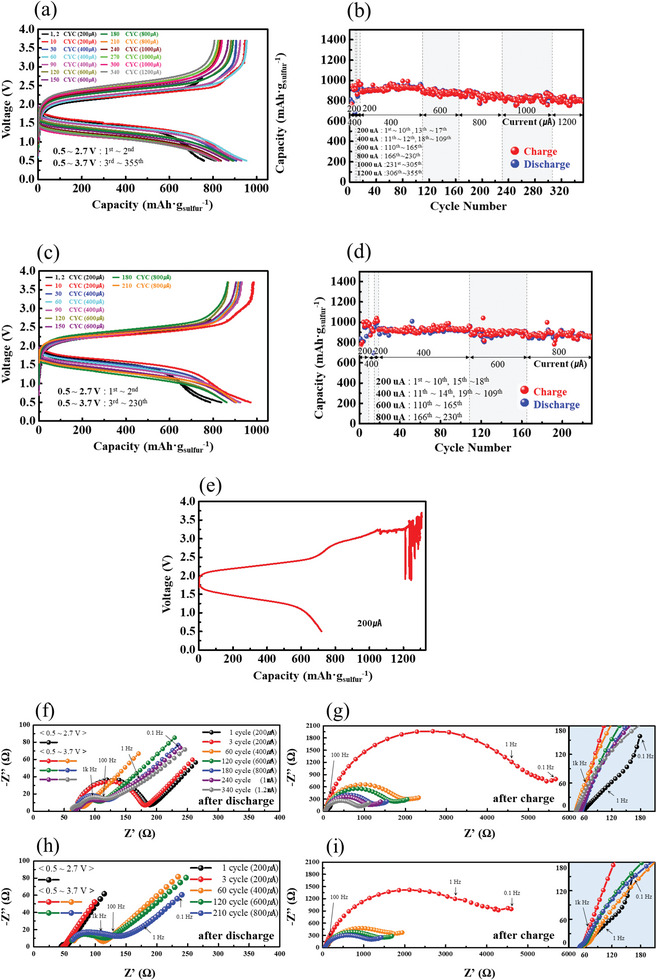
a) Discharge–charge curves and b) the corresponding discharge–charge capacities as a function of the cycle number for the standard T1 cell. c) Discharge–charge curves and d) the corresponding discharge–charge capacities as a function of the cycle number for the standard T2 cell. e) Discharge–charge curves for the standard T0 cell. Impedance spectra of the standard T1 cell measured f) after discharge and g) after charge, during the cycling tests corresponding to (a) and (b). Impedance spectra of the standard T2 cell measured h) after discharge and i) after charge, during cycling tests corresponding to (c) and (d).

To confirm the long‐term stability of T1 and T2 cells against chemo‐mechanical failure, we prepared standard T1 and T2 cells without the RE. Note that these cells used a thinner SE (≈370 µm) than those of the three‐electrode cells, which should increase the possibility of mechanical failure. **Figure** **6**a,b shows the discharge–charge curves and capacities of the standard T1 cell during 355 cycles, and Figure 6c,d shows those of the standard T2 cell during 230 cycles. The first two cycle tests were conducted in the cell‐voltage range of 0.5–2.7 V, and then in the range of 0.5–3.7 V. Both T1 and T2 cells operated stably for up to more than 200 cycles without any voltage noise that signal micro‐short circuit. In sharp contrast, chemo‐mechanical failure (voltage noise) occurred in T0 cell upon charging to 3.7 V, as shown in Figure 6e. Otoyama et al. observed short circuit and cracks in the LPS SE of a Li|LPS|Li cell due to volume expansion in the reductive decomposition product of LPS.^[^ ^52^ ^]^ In our case, the reduction product Li_2_S (+Li_3_P) was also formed in the T1 cell but only during the mixing step before cell fabrication; therefore, volume expansion could not be an issue in this cell. Moreover, the reduction product may function as “a passivating layer” with electron‐insulating characteristics to prevent further decomposition.^[^ ^52^ ^]^ From this result, we can deduce that even under high‐cutoff‐voltage conditions (such as 0.5–3.7 V), LPS in the cathode may be reversibly oxidized and reduced, provided that alloying–de‐alloying is reversibly processed with an even distribution of reactive sites, as in the composite alloy anode. Figures 6f,g show the impedance spectra measured for T1 cell after discharge and after charge, respectively, during the cycling tests corresponding to Figures 6a,b. Figures 6h,i show the impedance spectra measured for T2 cell after discharge and after charge, respectively, during the cycling tests corresponding to Figures 6c,d. While T1 cell exhibited an increasing trend after the initial decreases in charge‐transfer resistance (semicircle) that was measured after discharging, T2 cell exhibited a steady increase from the beginning. This difference in the resistance changing behavior between T1 and T2 cells can be explained by the presence of the reduction product in the T1 cell. The increasing resistance in the later cycles is likely due to typical electrode degradation (morphological and microstructural changes) with cycling. As observed in Figure 5, while the non‐Ohmic resistances of T1 and T2 cells reached 4500–5500 Ω after the initial 3.7 V charge, which was due to LPS oxidation in the cathode, it continuously decreased with cycling, reaching 1000–2000 Ω. Although this decreasing behavior cannot be clearly rationalized, it can be deduced that the oxidation products were stabilized in subsequent cycles, passivating the LPS against further reaction.^[^ ^37^ ^]^ Notably, the total resistances of the T1 and T2 cells were approximately the same at 100th–200th cycles, indicating that the performances of the T1 and T2 cells eventually became similar.

**Figure 7:**
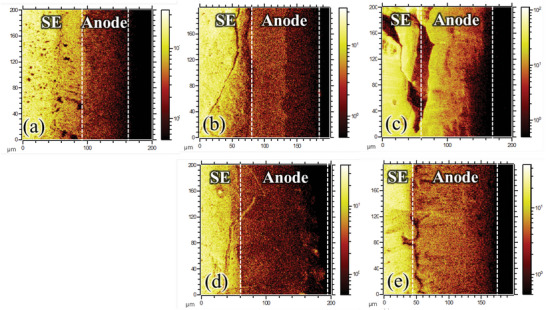
TOF‐SIMS data with respect to 7Li^+^ for T0 cell a) before tests, b) after 2.7 V charge, and c) after 3.7 V charge. TOF‐SIMS data with respect to 7Li^+^ for T1 cell d) after 2.7 V charge and e) after 3.7 V charge.

**Figure 8:**
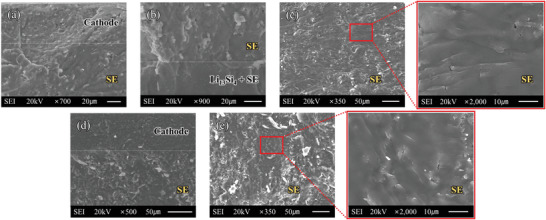
Cross‐sectional SEM images of a–c) T1 cell and d,e) T2 cell after cycling tests.

TOF‐SIMS analysis was carried out on the cross sections of T0 and T1 cells to characterize Li–Si alloying sites in the composite and non‐composite alloy anodes, after charging the cells to 2.7 and 3.7 V. **Figures** **7**a–c show the 7Li^+^ distribution for the T0 cell in the pristine state (before tests), after 2.7 V charge, and after 3.7 V charge, respectively. A brighter color indicates a higher ion concentration. Note that the relatively brighter SE region than the anode region is due to the matrix effects of different materials. In the pristine state, the 7Li^+^ fragment was well distributed through the Li–Si alloy anode with a thickness of ≈65 µm (Figure 7a). After first discharging to 0.5 V and then charging to 2.7 V, the anode thickness increased to ≈105 µm (Figure 7b). Furthermore, a bright region and a dark region were formed in the anode layer with a clear boundary between them, indicating that Li–Si alloying in the anode mainly occurred near the SE (hence the brighter color), whereas the other region was less alloyed or not alloyed at all, causing volume expansion. We deduce that the bright (dark) region had a higher (lower) Li ratio than that of Li_13_Si_4_ after the 2.7 V charge. Performance degradation was not observed in the T0 cell when cycling under the condition of 0.5–2.7 V despite the non‐uniform distribution of Li–Si alloying sites in this anode.^[^ ^54^ ^]^ However, such a non‐uniformity is by no means desirable in terms of long‐term cyclability. When charged to 3.7 V, Si in the anode was alloyed to reach a significantly higher Li ratio than Li_13_Si_4_, and Li metal might be plated because of LPS oxidation in the cathode. Such alloying and Li plating were concentrated in the anode region close to the SE, because, as shown in Figure 7c, the 7Li^+^ intensity in the anode is higher on the left side. Furthermore, more cracks and damage around the SE/anode interface are visible in Figure 7c compared with those in Figure 7a,b, which might be caused by severe over‐alloying and Li plating during 3.7 V charge. Figures 7d,e show the TOF‐SIMS data of T1 cell after 2.7 V charge and after 3.7 V charge, respectively. The brighter color in Figure 7e was due to over‐alloying and Li plating during the 3.7 V charge. Note that the dark/black colored region on the right side of the anode in Figure 7d might be attributed to the fractures that occurred during sample preparation. Compared with the T0 cell, the distribution of Li–Si alloying sites became more uniform in the T1 cell, and boundary separating bright and dark regions was not observed. In the designed composite structure, lithium‐ion conductivity was enhanced by providing more pathways for Li ions in the anode; therefore, non‐uniform alloying distribution and Li plating close to the SE (factors that eventually cause mechanical failure in SE) were effectively mitigated even when lithium ions were over‐supplied to the anode. The anode surface facing the anode current collector (stainless rod) was checked using a digital microscope. Figures S11a,b, Supporting Information, show the digital‐microscope images of the die‐pressed LiSi (1:1 ratio) and Li_13_Si_4_, respectively, from which we can observe that the color of Li_13_Si_4_ is more yellowish for a higher concentration of lithium. The Li_13_Si_4_ alloy anode (Type 0) was de‐alloyed (corresponding to the cell voltage of 0.5 V) and then alloyed (corresponding to the cell voltage of 3.7 V) in T0 cell. After the one‐cycle test, the cell was idle under open‐circuit conditions for 1 week, and then the anode surface facing the current collector was checked using a digital microscope, as shown in Figure S11c, Supporting Information. Some parts of the surface were accidently peeled off; therefore, we can observe the inner layer. The color of the surface and the inner layer appear different; the inner layer is more yellowish. The peeled‐off part is magnified at the right side of the figure. The results indicate that even after a long‐time rest lithium concentration gradient existed through the anode that was charged up to 3.7 V. **Figures** **8**a–e show the cross‐sectional SEM images of various locations of the tested T1 and T2 cells, respectively, after dissembling each cell from the mold. The T1 and T2 cells were tested for 38 and 59 cycles, respectively, and the corresponding discharge–charge curves of the T1 and T2 cells are shown in Figures S12a and S12b, Supporting Information, respectively. Note that a SEM image of the anode/SE interface of the T2 cell could not be obtained because most of the anode layer of T2 cells was missing. Although the cycling tests were short‐term conducted for post‐material analyses, both the cells were evidently crack‐free and free from indications of mechanical failure, in contrast with T0 cells (tested for only 17 cycles), which exhibited cracks and electroplating of Li metal in the SE and around the electrode interfaces.^[^ ^54^ ^]^

To summarize, the present study results demonstrate that the chemo‐mechanical stability of Li–S ASSBs could be achieved using alloy anodes with a composite structure, instead of Li metal foil, Li–In foil, or other types of Li‐based alloys without SE. Although LPS oxidation is inevitable in the sulfur‐composite cathode and its oxidative decomposition increases the polarization resistance during 3.7 V charging, this redox activity can be made reversible without causing a chemo‐mechanical failure, depending on the anode structure of the alloy. In addition, no substantial difference was observed between the electrochemical performance or stability of T1 and T2 cells, and Type 2 cell had no obvious advantage over Type 1 cell. The formation of Li_2_S and Li_3_P in the Type 1 composite anode is inevitable. Although this reaction might increase the initial polarization resistance of the anode, these products did not have a significant influence on the long‐term cyclability because the reduction products functioned as “a passivating layer” in the anode.^[^ ^58^ ^]^

## 3 Conclusion

In conclusion, two composite alloy anodes (Type 1: LPS glass + Li–Si alloy, Type 2: Li_3_N + LiF + Li–Si alloy) were prepared for use in sulfide‐based ASSBs, and their chemo‐mechanical stability under high‐cutoff–voltage conditions was compared with that of a non‐composite alloy anode. LPS glass and sulfur composites were selected as the SE and cathode, respectively. Type 1 and Type 2 cells were reversibly operated under high‐cutoff‐voltage conditions (0.5–3.7 V) without showing voltage noise or capacity fading for up to 355 and 230 cycles, respectively. The cathode polarization resistance drastically increased after 3.7 V charge owing to LPS oxidation; however, the LPS redox behavior was fairly reversible upon discharge–charge, unlike the cell using non‐composite alloy anode. TOF‐SIMS measurement results indicated that the composite anode achieved uniform Li–Si alloying by providing more pathways for lithium ions, even when the ions were over‐supplied via LPS oxidation in the cathode. These results imply that the LPS‐based cell can be reversibly cycled with the LPS redox under high‐cutoff‐voltage conditions, as long as non‐uniform alloying and lithium dendrite growth are avoided in the anode. No significant difference between the electrochemical performance and long‐term stability of T1 and T2 cells was observed. The reduction product Li_2_S was observed in T1 cell, but this might function as a passivating layer to prevent continuous reactions. Our study reveals the importance of the alloy anode structure with regard to chemo‐mechanical failure when the cell is cycled outside the ESW. The results also indicate that the ESW of sulfide SEs needs to be re‐evaluated for each practical application.

## 4 Experimental Section

LPS glass SE was prepared using planetary ball milling. Li_2_S (Aldrich, 98%) and P_2_S_5_ (Aldrich, 98%) in a molar ratio of 75:25 were placed in a zirconia pot and subjected to planetary ball milling (Fritsch Pulverisette 7, 370 rpm, 30 h). The sulfur‐composite cathode was prepared by ball milling a powder mixture of sulfur (Alfa Aesar, 325 mesh, 99.5%), acetylene black (MTI Corp, 99.9%), and the LPS glass in a weight ratio of 25:25:50 (Fritsch Pulverisette7, 370 rpm, 3 h). To prepare the composite anode, Li granules (Aldrich, 99%) and Si (Acros Organics, 325 mesh, 99%) were subjected to planetary ball milling (370 rpm, 2.5 h) to obtain a Li–Si alloy with a composition of Li_13_Si_4_. For the Type 1 composite anode, the Li–Si alloy powder and LPS glass SE were mixed at 80:20 w/w and ball‐milled (370 rpm, 2.5 h). For the Type 2 composite anode, a powder mixture of Li_3_N+LiF (in 75:25 molar ratio), instead of LPS SE, was subjected to ball milling (500 rpm, 72 h). Subsequently, the Li–Si alloy and Li_3_N+LiF powders were mixed at 80:20 w/w and ball‐milled again (370 rpm, 2.5 h). Detailed information on fabricating the Li–Si alloy, sulfur composite cathode, and LPS glass SE could be found in previous studies.^[^ ^61^, ^64^ ^]^ XRD analysis (PANalytical, X'Pert PRO MPD) was performed on the prepared LPS glass SE, composite anode (Type 1 and Type 2), and composite cathode powders.

To fabricate the standard cells, three layers (SE: ≈64.9 mg cm^−2^, composite anode of Type 1 or 2: ≈8.11 mg cm^−2^, and composite cathode: ≈12.99 mg cm^−2^) were successively and uniaxially pressed under 330 MPa using alumina molds and stainless‐steel rods. The active (electrode) area of the cell was ≈1.54 cm^2^. Note that the weights of the cathode active material (sulfur) and anode active material (Li–Si alloy) were fixed for all the cells in this study. Figure 3a,b shows the schematics of the two‐electrode (standard) cells with Type 1 and 2 composite anodes (denoted as T1 and T2 cells, respectively). The three‐electrode cells were prepared to allow the separation of the cathode and anode potentials, and to separately trace their polarization resistances. Indium (In) metal was employed as the reference electrode (RE) in these cells because Li–In alloy was more compatible (chemically stable) with sulfide SEs than Li metal,^[^ ^62^, ^65^, ^66^, ^67^ ^]^ and Li*_x_* In also shows a constant electrode potential of ≈0.62 V versus Li/Li^+^ in the range of 0 < *x* ≤ 0.45.^[^ ^68^ ^]^ Specifically, a strip of In metal foil (13 mm × 133 mm × 0.127 mm, measured before pressing) was used as the embedded RE in the middle of the SE layer. Detailed information on fabricating the three‐electrode ASSBs can be found in the authors’ previous study.^[^ ^69^ ^]^ A wire‐shaped RE was selected to minimize current distortion and artificial effects.^[^ ^70^ ^]^ The corresponding schematics of the three‐electrode T1 and T2 cells are shown in Figures 3c,d, respectively. A non‐composite anode (Li–Si without any SE, denoted as Type 0 anode) was also prepared for comparison, and the corresponding reference cells (denoted as T0 cell) were prepared under identical conditions to T1 and T2 cells. Before electrochemical tests, the microstructure and elemental distribution in the constructed cells were analyzed using SEM (ZEISS, Gemini‐500) with EDS (Oxford, Ultim‐MAX 10).

Cycling tests were conducted on the standard T1 and T2 cells under a constant current of 200–1200 µA (0.13–0.78 mA cm^−2^) and 200–800 µA (0.13–0.52 mA cm^−2^), respectively. For the first two cycles, the cells were cycled between 0.5 and 2.7 V, and then the cutoff–voltage condition was changed to 0.5–3.7 V. Both the voltage ranges were outside the thermodynamically predicted ESW of LPS (1.7–2.1 V vs Li/Li^+^). The gravimetric capacities measured in the present study were based on the weight of the cathode active material, namely sulfur. Before cycling tests on the three‐electrode cells, the embedded In RE was lithiated by supplying a constant current of 20 µA for 40 min between the RE and anode to form the Li*_x_* In alloy via a two‐phase reaction In + Li^+^ + e^−^ → LiIn. The cathode–anode, cathode–RE, and RE–anode voltages (denoted as V1, V2, and V3, respectively, see Figure 1) were monitored during discharge–charge under the cutoff‐voltage conditions of 0.5–2.7 V and then 0.5–3.7 V. Additionally, the impedance spectra of the full cell (cathode–anode) and two half‐cells (cathode–RE and anode‐RE) were separately and frequently measured on the three‐electrode cell during the tests. All the electrochemical tests were conducted using a potentiostat/galvanostat using an impedance analyzer (Bio‐Logic SP‐240). Cell fabrication and testing were performed in a glove box filled with Ar, with both the oxygen and water concentrations maintained below 1 ppm.

After conducting the tests, cross sections of the standard T0 and T1 cells were machined using IMS (ArBlade 500, Hitachi High‐Tech) and examined using TOF‐SIMS (TOFSIMS5, IONTOF) to characterize and compare the Li distribution in Type 0 and Type 1 anodes at different states of charge (2.7 and 3.7 V). The sample surface was refreshed using cesium‐ion beam sputtering with a raster size of 500 µm for 1 min. TOF‐SIMS measurements were performed using Bi_3_^+^ with an energy of 30 keV as the primary ion source. During cycling tests at 0.5–3.7 V, cross sections of the tested T1 and T2 cells were examined using SEM for possible formation of cracks and other types of mechanical failure in the SE layer.

## Conflict of Interest

The authors declare no conflict of interest.

## Supporting information

Supporting Information

## Data Availability

The data that support the findings of this study are available from the corresponding author upon reasonable request.
