# Supplementary material for: Design Strategies of Li–Si Alloy Anode for Mitigating Chemo‐Mechanical Degradation in Sulfide‐Based All‐Solid‐State Batteries
Source: Adv Sci (Weinh). 2023 Jun 26;10(24):2301381. doi: 10.1002/advs.202301381 (PMC10460900; doi:10.1002/advs.202301381)
Supplement: Supplementary file 1 — Supporting Information [file ADVS-10-2301381-s001.pdf]

ADVANCED SCIENCE
Open Access

Supporting Information

for *Adv. Sci.*, DOI 10.1002/advs.202301381

Design Strategies of Li–Si Alloy Anode for Mitigating Chemo-Mechanical Degradation in Sulfide-Based All-Solid-State Batteries

*Minhyung Kim, Min Ju Kim, Yeong Seon Oh, Sung Kang, Tae Ho Shin and Hyung-Tae Lim**

Supporting Information

Design Strategies of Li–Si Alloy Anode for Mitigating Chemo-Mechanical Degradation in Sulfide-Based All-Solid-State Batteries

*Minhyung Kim, Min Ju Kim, Yeong Seon Oh, Sung Kang, Hae To Shin, Hyung-Tae Lim**

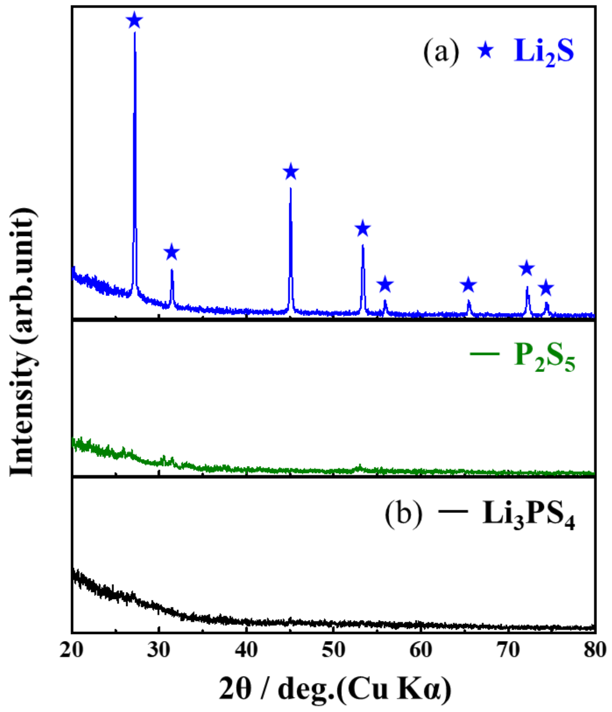

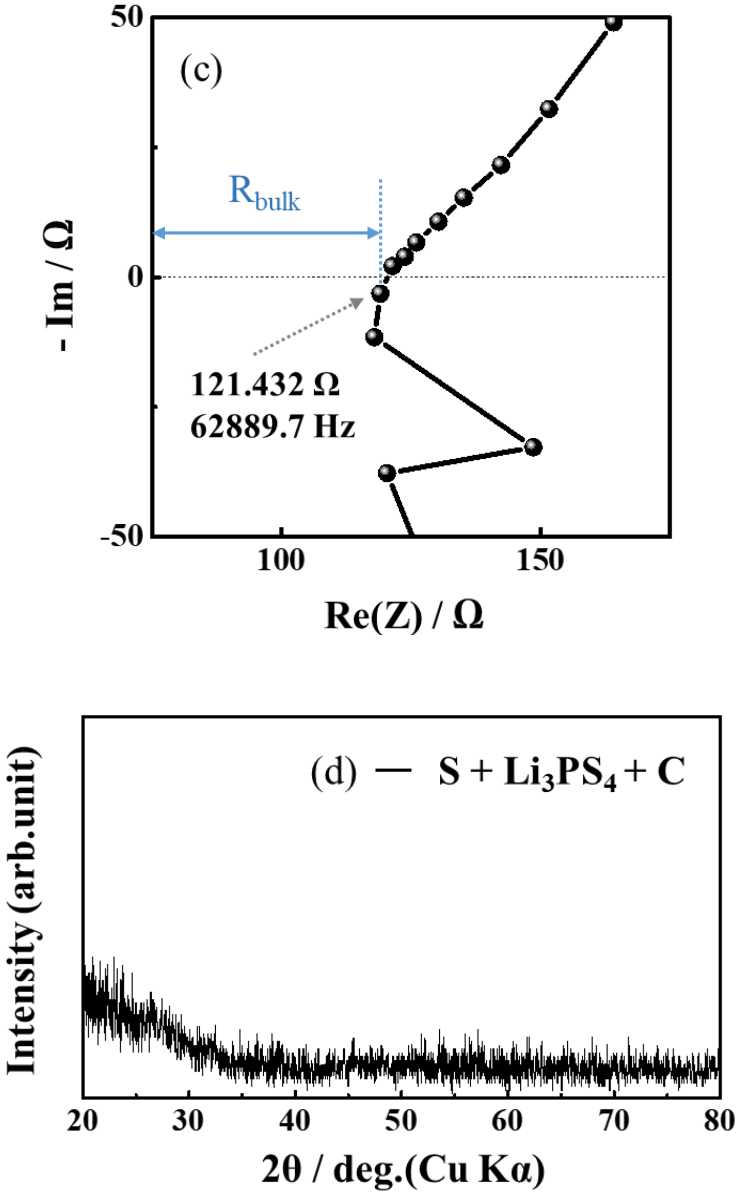

Figure S1. XRD patterns of mixed Li_2S and P_2S_5 powder (a) before and (b) after planetary-ball milling at 370 rpm for 30 h. (c) Impedance spectra of LPS glass SE. (d) XRD pattern of the composite cathode powder after ball milling.

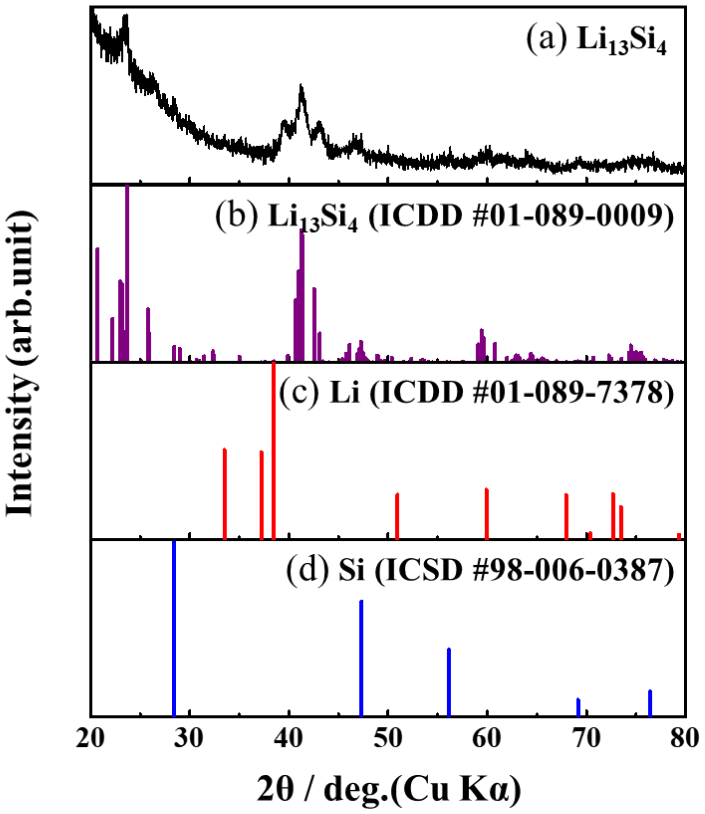

Figure S2. XRD patterns of (a) $Li_{13}Si_4$ alloy anode powder obtained after mechanical alloying, (b) $Li_{13}Si_4$, (c) Li, and (d). Patterns (b–d) are from the International Centre for Diffraction Data (ICDD) database.

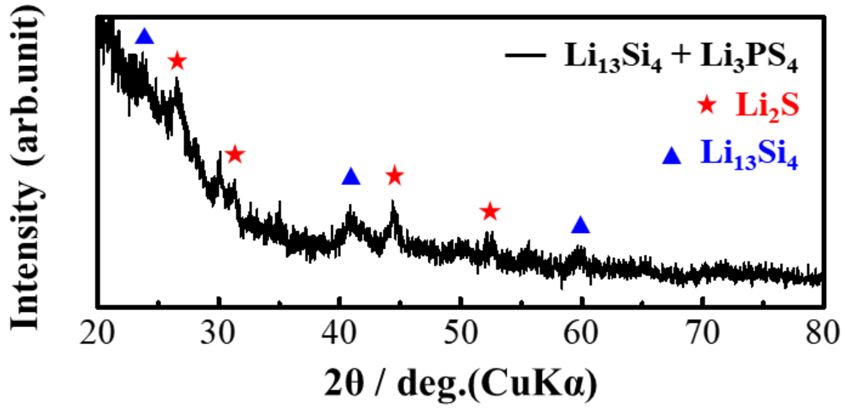

Figure S3. XRD pattern of Type 1 composite anode powder after ball milling.

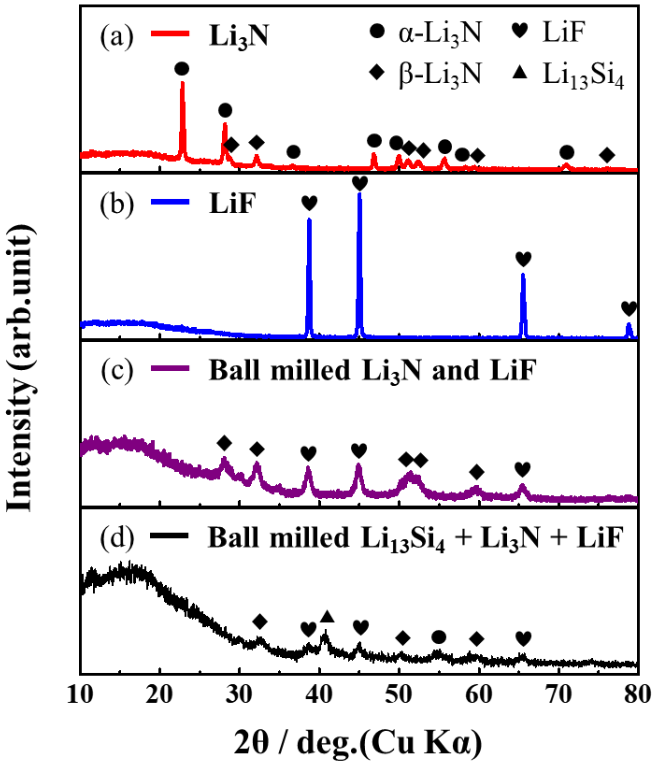

Figure S4. XRD patterns of (a) as-received Li_3N, (b) as-received LiF, (c) ball-milled composite powder of Li_3N and LiF, and (d) ball-milled Type 2 composite anode powder.

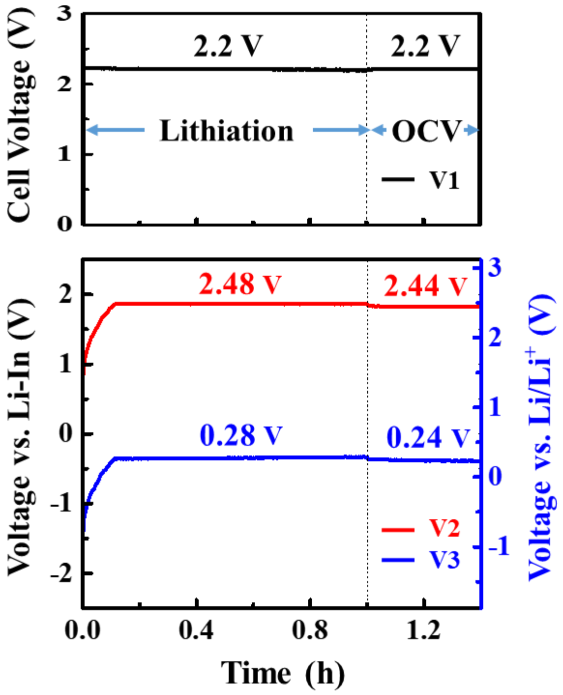

Figure S5. Voltage profiles of V1 (black), V2 (red), and V3 (blue) for the three-electrode T0 cell during lithiation of RE (0–1 h) and then under open-circuit conditions after lithiation (1–1.2 h).

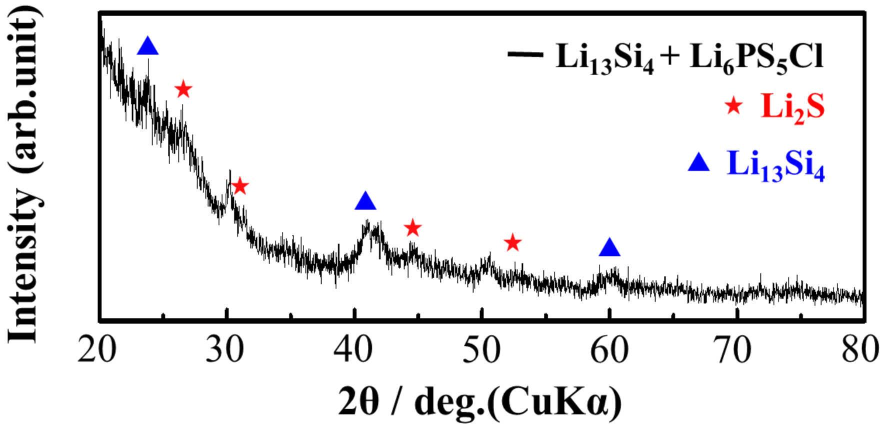

Figure S6. XRD pattern of the composite powder of Li_6PS_5Cl and $Li_{13}Si_4$ after ball milling.

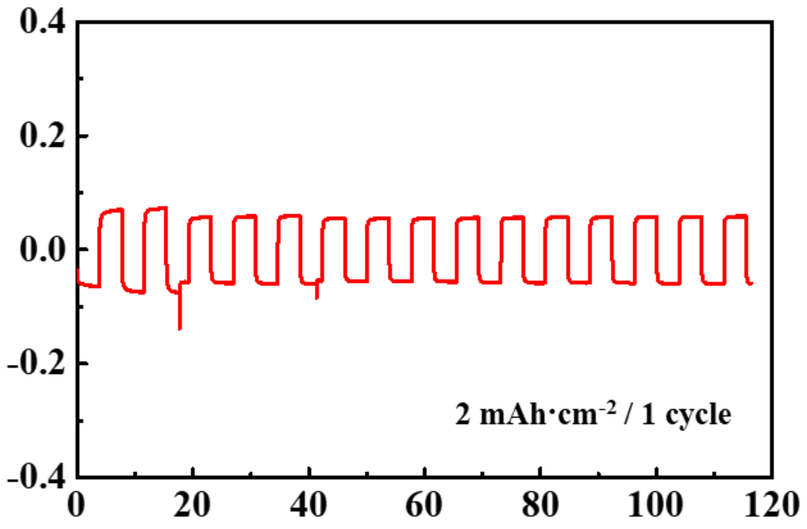

Figure S7. Discharge–charge curves of a cell consisting of (Li_7Si_3 + LPS) | LPS | ($Li_{13}Si_4$+LPS) before the initial lithiation of the (Li_7Si_3 + LPS) electrode at 0.26 mA cm^{-2} at 2 mAh cm^{-2} per cycle.

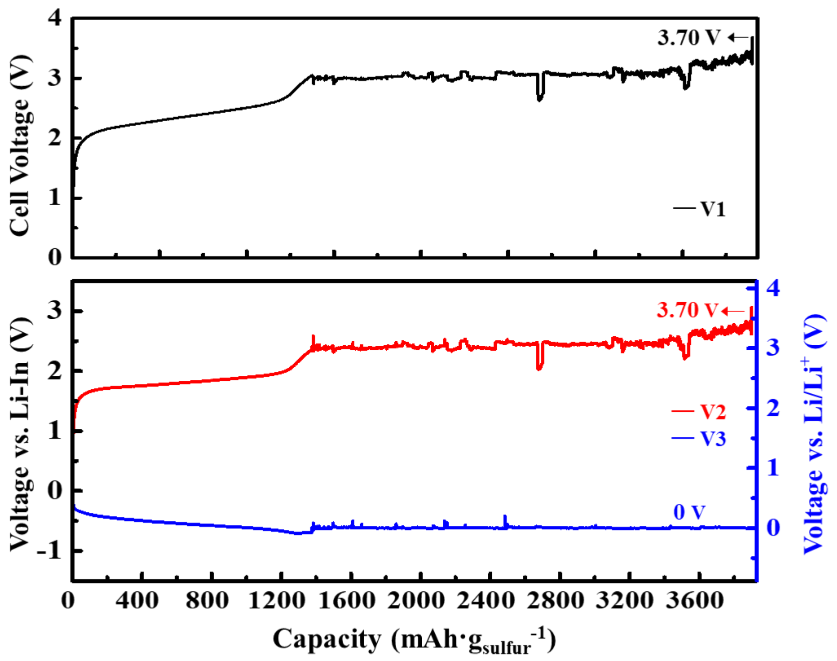

Figure S8. Voltage profiles of V1, V2, and V3 for T0 cell during 8th 3.7 V charge. Note that 1st and 2nd cycles were conducted in the cell voltage range of 0.5–2.7 V, and 3rd - 17th (final) cycles were conducted in 0.5–3.7 V. Reproduced with permission.[54] Copyright 2022, Elsevier.

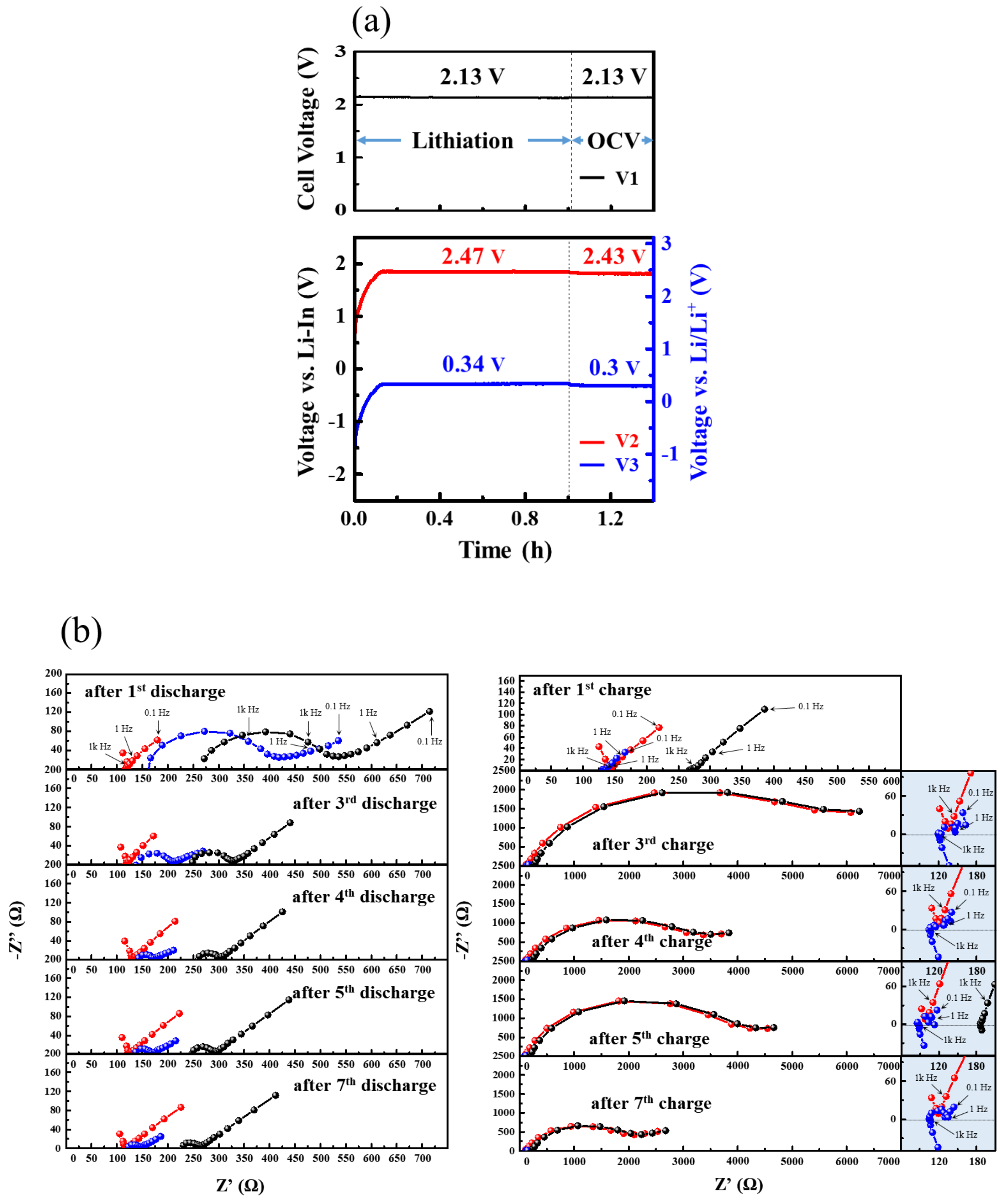

Figure S9. (a) Voltage profiles of V1, V2, and V3 for the three-electrode T1-1 cell during the lithiation of RE and then under open-circuit conditions after the lithiation. (b) Impedance spectra of T1-1 cell during the discharge–charge tests. Note that the first two cycles were conducted in the cell voltage range of 0.5–2.7 V, and the other cycles in 0.5–3.7 V range.

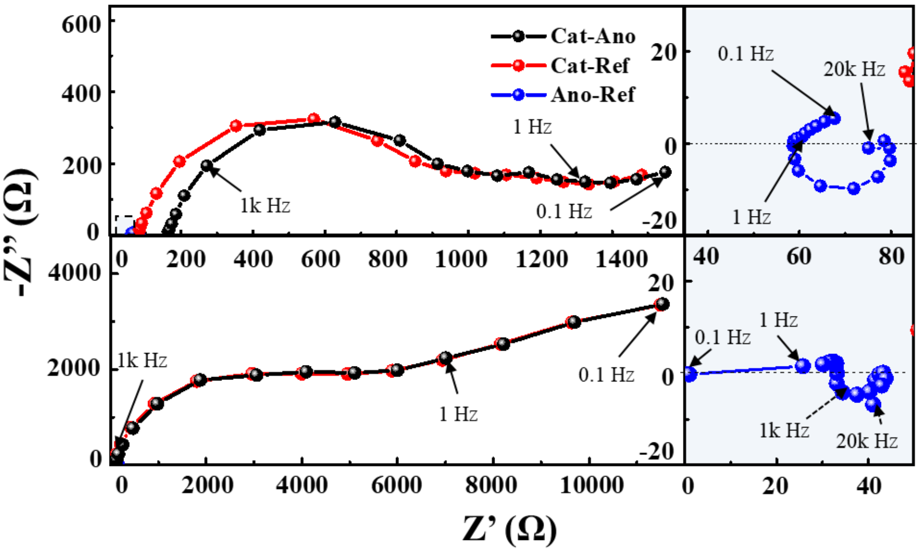

Figure S10. Impedance spectra of the three-electrode T0 cell measured during the discharge–charge tests; after 17th discharge to 0.5 V (top) and after 17th charge to 3.7 V (bottom). Note that 1st and 2nd cycles were conducted in the cell-voltage range of 0.5–2.7 V, and 3rd–17th (final) cycles in 0.5–3.7 V range. Reproduced with permission. [54] Copyright 2022, Elsevier.

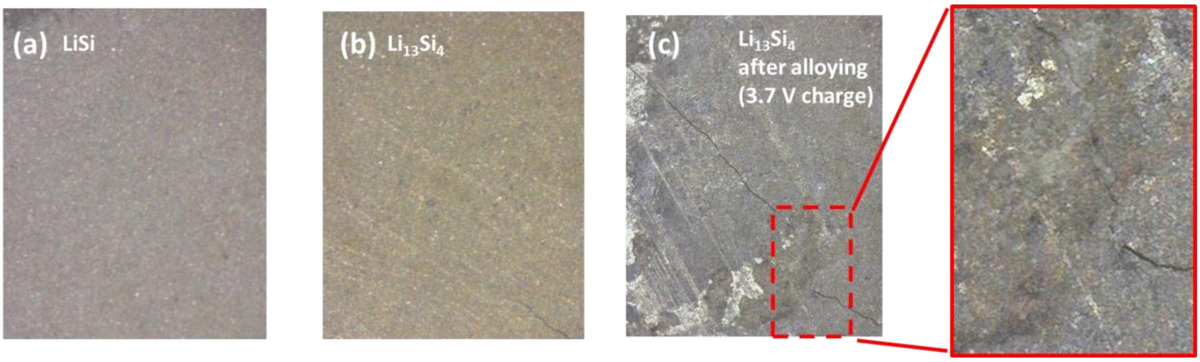

Figure S11. Digital-microscope images of (a) LiSi (1:1 ratio) and (b) $Li_{13}Si_4$ after die-pressing; (c) digital-microscope image of the surface of $Li_{13}Si_4$ (Type 0 anode) of T0 cell facing the current collector after 3.7 V charge. The peeled-off part is magnified in a red box.

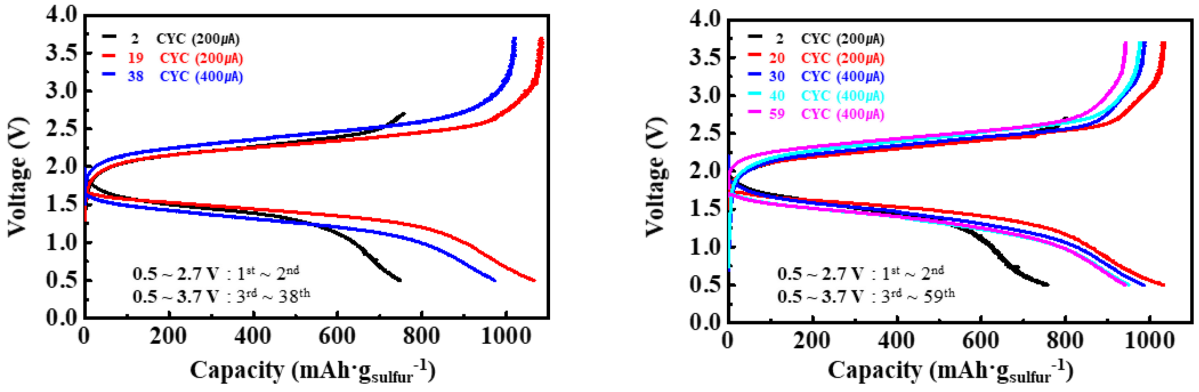

Figure S12. Discharge–charge curves of (a) T1 and (b) T2 cell corresponding to Figure 7a–c and d–e, respectively.
